# The effect and underlying mechanism of yeast β-glucan on antiviral resistance of zebrafish against spring viremia of carp virus infection

**DOI:** 10.3389/fimmu.2022.1031962

**Published:** 2022-11-03

**Authors:** Hui Liang, Yu Li, Ming Li, Wei Zhou, Jie Chen, Zhen Zhang, Yalin Yang, Chao Ran, Zhigang Zhou

**Affiliations:** ^1^ Key Laboratory for Feed Biotechnology of the Ministry of Agriculture and Rural Affairs, Institute of Feed Research, Chinese Academy of Agricultural Sciences, Beijing, China; ^2^ Laboratory of Gene Therapy, Department of Biochemistry, College of Life Sciences, Shaanxi Normal University, Xi’an, China; ^3^ Sino-Norway Joint Lab on Fish Gut Microbiota, Institute of Feed Research, Chinese Academy of Agricultural Sciences, Beijing, China

**Keywords:** β-glucan, zebrafish, SVCV, antiviral immunity, gut microbiota

## Abstract

β-glucan has been used as immunostimulant for fish. However, the effect of yeast β-glucan on viral infections has been less studied in fish. In this study, we investigated the effects of β-glucan on the resistance of zebrafish against spring viraemia of carp virus (SVCV) and elucidated the underlying mechanisms. Zebrafish were fed with a control diet or diet supplemented with 0.01% and 0.025% β-glucan for 2 weeks, and were challenged by SVCV. Zebrafish embryonic fibroblast (ZF4) cells were treated with 5 μg/mL β-glucan and were infected by SVCV. We further investigated the effect of β-glucan on autophagy level post SVCV infection. The intestinal microbiota was evaluated by 16S *r*RNA gene pyrosequencing. Results showed that dietary supplementation of 0.025% β-glucan significantly increased survival rate of zebrafish compared with control group after SVCV challenge (*P* < 0.05). Dietary β-glucan significantly increased the expression of genes related to type I IFN antiviral immune pathway in the spleen of zebrafish after viral infection, including type I IFN genes (*ifnφ1*, *ifnφ2*, *ifnφ3*), IFN-stimulated genes (*mxb*, *mxc*), as well as other genes involved in the IFN signaling pathway, including *tlr7*, *rig1*, *mavs*, *irf3* and *irf7*. Morpholino knockdown of type I IFN receptors dampened the antiviral effect of β-glucan in zebrafish larvae, indicating that β-glucan-mediated antiviral function was at least partially dependent on IFN immune response. Furthermore, β-glucan can inhibit the replication of SVCV in ZF4 cells. However, β-glucan did not stimulate type I IFN antiviral response in ZF4 cells, and the antiviral effect of β-glucan in ZF4 was independent of Myd88. Interestingly, β-glucan induced autophagy in ZF4 cells after SVCV infection. Inhibition of autophagy blocked the antiviral effect of β-glucan in ZF4 cells. Lastly, dietary β-glucan changed the composition of intestinal microbiota in zebrafish, with reduced abundance of Proteobacteria and an enrichment of Fusobacteria and Firmicutes. To sum up, our results indicate that the β-glucan enhanced resistance of zebrafish against SVCV and the mechanism involved stimulation of type I IFN antiviral immune response of fish after viral infection.

## Introduction

Yeast cell wall consists of β-glucan, mannan, protein, lipid, and chitin, among which the proportion of β -glucan was the highest, about 29% to 64% (1, 2). Previous studies have shown that β-glucan is a natural immune activator that can nonspecifically and specifically enhance the immune system of aquatic animals (3–6). Early studies discovered that β-glucan from yeast cell wall can act on macrophages to stimulate the body’s non-specific immunity (7). β-Glucan can be administered by different routes such as injection, bathing, and dietary supplementation, and the beneficial effects of β-glucan have been reported in various fish species including Atlantic salmon (*Salmo salar*), common carp (*Cyprinus carpio*), Nile tilapia (*Oreochromis niloticus*), etc (8). As a key facet of the benefits, β-glucan can enhance the host’s defenses to improve their resistance to pathogens. For instance, macrophages from β-glucan-treated rainbow trout exhibited increased bactericidal activity against *Aeromonas salmonicida* (9). Yeast β-glucan was also used as vaccine adjuvant to protect fish against various bacterial pathogens, including *Vibrio damsela* (10), *Edwardsiella tarda* (11). In contrast to bacterial diseases, the effect of yeast β-glucan on viral infections has been less studied in fish. Eearly study showed that administration of β-glucans to rainbow trout results in decreased susceptibility to IHNV, presumably as a result of an enhanced innate response (12). β-Glucan treatment improved survival of Olive flounder (*Paralichthys olivaceus*) after challenge by Viral hemorrhagic septicemia (VHS) (13). Beaulaurier et al. showed that Pacific herring (*C. pallasii*) fed a β-glucan-containing diet significantly improved survival when challenged with viral haemorrhagic septicaemia rhabdovirus (VHSV) (14). Carp treated with glucan showed enhanced resistance against the infection of grass carp hemorrhage virus (GCHV) (15). Krishnan et al. revealed that β-glucan protected grouper against NNV infection (16). Till now, the mechanism of the antiviral effect of β-glucan has been poorly understood.

Spring viremia of carp (SVC) is caused by spring viremia of carp virus (SVCV) and leads to mass cyprinid mortality and enormous economic losses. In host cells, viral RNAs can be recognized by the innate antiviral immune system, triggering an antiviral response (17). Until now, there is no licensed therapeutic agents available for controlling SVCV infection (18). Type I interferon (IFN) system plays an important role in the antiviral immune response of fish (19). Consistent with the function in mammals, type I IFN in fish can directly inhibit the replication of the virus by inducing the expression of antiviral proteins (19–21). It has been reported that β-glucan can enhance the immune response and resistance of zebrafish against SVCV infection (22). However, the response of type I IFN signaling pathway was less investigated in glucan-treated fish after SVCV infection.

Autophagy is a self-protection ability acquired by the body in long-term evolution. Autophagy can non-specifically degrade pathogenic microorganisms such as bacteria, viruses, and parasites that invade the host (23). Recent studies have shown that autophagy has a double-sided effect on viral infection. On the one hand, autophagy can clear viruses; on the other hand, some viruses can effectively complete their own replication by using autophagy (24). For example, when Sindbis virus invades host cells, autophagy can be induced, and host cells clear the virus through autophagy (25). Infection with vesicular stomatitis virus (VSV) causes autophagy in *Drosophila*, which in turn inhibits viral replication (26). In contrast, autophagy promoted the replication of polioviruses (27) and dengue virus (28). In terms of fish viruses, previous studies showed that autophagy inhibited the replication of two fish rhabdoviruses, viral hemorrhagic septicemia virus (VHSV) and SVCV in zebrafish embryonic fibroblast (ZF4) cells (29, 30). However, studies also showed that SVCV utilizes the autophagy pathway to promote viral replication in Epithelioma papulosum cyprini (EPC) cells (31). The discrepancy in terms of the effect of autophagy on SVCV infection might be due to different cell models used.

In the present study, we evaluated the effect of dietary β-glucan on antiviral resistance in zebrafish through SVCV infection model, and investigated the underlying mechanisms. Our results showed that dietary β-glucan enhanced the resistance of zebrafish against SVCV infection. Moreover, we found that β-glucan stimulated the antiviral IFN response in zebrafish, and the antiviral effect of β-glucan involved autophagy induction.

## Materials and methods

### β-Glucan and experimental diets

Two types of diets were prepared, the basal diet and β-glucan supplemented diet. The β-glucan was obtained from Solarbio (Beijing, China: purity ≥ 80.0%). The composition of basal diet is shown in **Table 1**. β-Glucan supplemented diet was formulated by adding 0.01% and 0.025% β-glucan into basal diet, which replaced the same amount of bentonite. β-Glucan was dissolved in appropriate amount of sterile water and then mixed with basal diet.

**Table 1:** Ingredients and proximate compositions of the experimental diets (g/100 g dry diet).

Ingredient	Control	0.01% β-glucan	0.025% β-glucan
Casein	40	40	40
Gelatin	10	10	10
Dextrin	28	28	28
β-Glucan	0	0.01	0.025
Soybean oil	6	6	6
Lysine	0.33	0.33	0.33
VC phosphate ester	0.1	0.1	0.1
Vitamin mix ^a^	0.4	0.4	0.4
Mineral mix ^b^	0.4	0.4	0.4
CaH_2_PO_4_	2	2	2
Choline chloride	0.2	0.2	0.2
Sodium alginate	2	2	2
Microcrystalline cellulose	4	4	4
Zeolite	6.57	6.56	6.545
Total	100	100	100
Crude protein ^c^	42.19	42.19	42.19
Crude fat ^c^	6.09	6.09	6.09
Total energy (KJ/g DM) ^c^	18.55	18.55	18.55

a : Containing the following (g/kg vitamin premix): thiamine, 0.438; riboflavin, 0.632; pyridoxine·HCl, 0.908; d-pantothenic acid, 1.724; nicotinic acid, 4.583; biotin, 0.211; folic acid, 0.549; vitamin B-12, 0.001; inositol, 21.053; menadione sodium bisulfite, 0.889; retinyl acetate, 0.677; cholecalciferol, 0.116; dl-α-tocopherol-acetate, 12.632.

b : Containing the following (g/kg mineral premix): CoCl_2_·6H_2_O, 0.074; CuSO_4_·5H_2_O, 2.5; FeSO_4_·7H_2_O, 73.2; NaCl, 40.0; MgSO_4_·7H_2_O, 284.0; MnSO_4_·H_2_O, 6.50; KI, 0.68; Na_2_SeO_3_, 0.10; ZnSO_4_·7H_2_O, 131.93; Cellulose, 501.09.

c : Total energy, crude protein and crude fat was calculated by excel software.

### Fish husbandry

For feeding experiments, we used 2-months-old Tuebingen zebrafish obtained from the China Zebrafish Resource Center (Wuhan, China), with an average initial weight of 0.08 ± 0.005 g. The zebrafish were randomly assigned to 3 L tanks each containing 15 fish, with 3 replicate tanks per group. During 2-week feeding period, zebrafish were fed twice (09:00 and 17:00) a day, with 6% of the total body weight. The water temperature 28°C, pH 6.7-7.0, O_2_ > 6.0 mg/L, and nitrogen content < 0.50 mg/L. For the experiments with zebrafish larvae, larvae hatched from their chorions at 3 days postfertilization (dpf). Each group had four bottles with 30 fish per bottle. At 4 dpf, the zebrafish larvae were treated with β-glucan by immersion at 0.025% and 0.05% (*m*/*v*). At 7 dpf, 0.1 MOI SVCV was added. At 9 dpf, zebrafish larvae were collected for *q*PCR analysis.

### SVCV challenge and the 50% tissue culture infectious dose assay

Zebrafish from each experimental group were acclimatized to 22°C during the last week of feeding and then challenged with 10^6^ TCID_50_/ml SVCV by bath immersion. Zebrafish were not fed during the challenge period and the mortality was monitored daily for 14 days. The survival rate was calculated by the Kaplan-Meier method. Zebrafish were anesthetized with MS222, then the spleen was sampled and RNA was extracted, and the expression of antiviral genes of zebrafish was detected.

### Cells culture and virus propagation

ZF4 cells were purchased from the American Type Culture Collection (ATCC number CRL-2050), and cultured in DMEM/F-12 medium containing 10% fatal bovine serum (Gibco, Australia) at 28°C in a 5% CO_2_ incubator. EPC cells and SVCV (ATCC: VR-1390) were presented by professor Jun-Fa Yuan (Huazhong agricultural university, Wuhan, Hubei, China). EPC cells in MEM medium at 25°C in an incubator with 5% CO_2_. The SVCV was propagated in EPC cells at 28°C. When cytopathic effect (CPE) was clearly visible in EPC cells, the viral suspensions were divided into aliquots and stored at -80°C.

### Cell survival rate analysis

ZF4 cell was seeded on 96-well plates and incubated for 24 h to sub-confluence. Then ZF4 cell was exposed to medium added with β-glucan. At the end of the exposure period, medium with 10% AlarmaBlue cell viability reagent (Invitrogen, Grand Island, NY, USA) was added. After a 1 h incubation, fluorescence was measured with the SynergyH1 microplate reader (Biotek) at excitation and emission wavelengths of 485 nm and 595 nm, respectively. The ratio of cell viability was calculated using the fluorescence readings. Cell survival rate = (value of treatment group - value of blank control group)/(OD value of negative control group - OD value of blank control group) ×100%.

### Detection of antiviral effect of β-glucan in ZF4 cells

ZF4 cells were treated with β-glucan (1, 2.5, 5, 10, 20 μg/mL) for 24 h. Then 0.1 MOI SVCV was added and incubated at 22°C for 1 h. Infected ZF4 cells monolayers were then washed, fresh medium added, and plates further incubated. At 24 hours after infection, SVCV replication in ZF4 cells was evaluated by *q*PCR measuring the expression of SVCV N protein. ZF4 cells infected with SVCV but not treated with β-glucan were used as control. For TCID_50_ assay, EPC cells were maintained in 96 well plates. The supernatants of ZF4 cells were collected at 24 h post SVCV infection. Then, serial 10-fold dilutions of supernatants were performed to incubate with EPC cells. After five days, the wells with CPE were recorded and TCID_50_/0.1 ml was calculated by the Reed-Muench method (32).

### Myd88 gene silencing with small interfering RNA

In a 12-well plate, ZF4 cells in the logarithmic growth phase were inoculated. The reagent Lipofectamine RNAiMAX Transfection (Invitrogen) was used to transfect scrambled small interfering RNA (siRNA; negative control) and *myd88* siRNA into the plate according to the kit procedure. The sequence of *myd88* siRNA is as follows: Sense: GGUCAUCUCUGAUGAUUAUTT; Antisense: AUAAUCAUCAGAGAUG ACCTT. After 24 h, β-glucan was added. SVCV at a concentration of 0.1 MOI was administered after 6 h. *q*PCR was used to determine the knockdown efficacy of the siRNA as well as the expression of the SVCV N protein.

### Morpholino knockdown

Vivo-morpholino oligonucleotides (MO) against zebrafish type I IFN receptor subunits CRFB1 and CRFB2 (33) were designed and synthesized by Gene-Tools (Philomath, OR). The sequences of MO used in this study are as follows: CRFB1-vivo-MO (splice blocking), 5′-CGCCAAGATCATACCTGTAAAGTAA-3′; CRFB2-vivo-MO (splice blocking), 5′-AGTTTGTTTTCTCACCTCTGTTCCA-3′; and standard control-vivo-MO, 5′-CCTCTTACCTCAGTTACAATTTATA-3′. Zebrafish larvae (4dpf) were added with 25 nmol/L of CRFB1 and CRFB2 vivo MO (CRFB1 and CRFB2 vivo MO were added together during the experiment), or standard control vivo MO, and then treated with 0.05% β-glucan for 24 h. At 7 dpf, 0.1 MOI SVCV was added. At 9 dpf, zebrafish larvae were collected and SVCV replication in the larvae was measured by qPCR. The knockdown efficiency was evaluated by qPCR quantifying the relative expression of properly spliced transcripts. Related primers are listed in **Table 2**.

**Table 2:** Primer sequences for *q*RT-PCR analysis.

Gene	Forward primer (5’-3’)	Reverse primer (5’-3’)	Accession No.
*rps11*	ACAGAAATGCCCCTTCACTG	GCCTCTTCTCAAAACGGTTG	NM_213377.1
*svcv n*	TGAGGTGAGTGCTGAGGATG	CCATCAGCAAAGTCCGGTAT	NC_002823
*svcv g*	TACAGATTCGGGGGATCTTG	ACCAACGTTCCATCAACACA	U18101.2
*ifnφ1*	GAGCACATGAACTCGGTGAA	TGCGTATCTTGCCACACATT	NM_207640.1
*ifnφ2*	CCTCTTTGCCAACGACAGTT	CGGTTCCTTGAGCTCTCATC	NM_001111082.1
*ifnφ3*	TTCTGCTTTGTGCAGGTTTG	GGTATAGAAACGCGGTCGTC	NM_001111083.1
*mxb*	AATGGTGATCCGCTATCTGC	TCTGGCGGCTCAGTAAGTTT	NM_001128672.1
*mxc*	GAGGCTTCACTTGGCAACTC	TTGTTCCAATAAGGCCAAGC	NM_001007284.2
*rig1*	TTGAGGAGCTGCATGAACAC	CCGCTTGAATCTCCTCAGAC	NM_001306095.1
*irf3*	CAAAACCGCTGTTCGTGCC	CATCGTCGCTGTTGGAGTCCT	NM_001143904.1
*irf 7*	AGGCAGTTCAACGTCAGCTACCAT	TTCCACCAAGTTGAGCAATTCCAG	NM_200677.2
*tlr7*	GGGAGTTTCAGGACAGCCTT	TTCCTTGGCCACTCCAAAACT	XM_021479060.1
*il1β*	GGCTGTGTGTTTGGGAATCT	TGATAAACCAACCGGGACA	NM_212844.2
*il10*	ATAGGATGTTGCTGGGTTGG	GTGGATGAAGTCCATTTGTGC	NM_001020785.2
*tnfα*	GCGCTTTTCTGAATCCTACG	TGCCCAGTCTGTCTCCTTCT	NM_212859.2
*mavs*	GTTCCCGGTCCAAGACACTA	TTGTCGCCTGAGTTGTTCTG	NM_001327873.1
*crfb1*	CATCATACACTGGCCGTCTT	TGTCTGAAGTTGTGAGACCATATT	XM_021478750.1
*crfb2*	TGGAACAGAGTTTACTGTGGATAAG	GTGGGCTTCGATCAATGATACT	XM_005167960.4

### Autophagy detection by AO staining and flow cytometry

ZF4 cells were treated with 5 μg/mL β-glucan when they reached 80% abundance, SVCV was added after 24 h, cells were trypsinized at 0 h, 6 h and 12 h after challenge. 1 mg AO (acridine orange) was dissolved in 10 mL of PBS with pH = 7.2 to prepare a 100 μg/mL stock solution. 100 μL of cell fluid was added to 4 μL of AO stock solution (acridine orange) staining solution. The fluorescence of stained cells was quantified by flow cytometry.

### Western blotting analysis

ZF4 cells were lysed with ice-cold RIPA lysis buffer mixed with 1mM PMSF and phosphatase inhibitors (Abcam, USA). Equivalent amounts of total protein were loaded into a 12% SDS-PAGE for electrophoresis and then transferred onto a polyvinylidene difluoride (PVDF) membrane (Millipore, USA). After blocking nonspecific binding with 5% non-fat dry milk in PBS, the PVDF membrane was incubated with primary antibodies, i.e., antibodies against β-actin (CMCTAG, AT0544, 1:1000) and LC3A/B (CST, 4108, 1:1000). The blots were developed using horseradish peroxidase (HRP)-conjugated secondary antibodies (GE Health, 1:3000) and the ECL-plus system.

### Autophagy inhibition

ZF4 cells were treated with autophagy specific inhibitor 3-methyladenine (3-MA) at 5 mM (MedChemExpress) and chloroquine (CQ) at 5 μM (MedChemExpress) for 24 h, and then treated with 5 μg/mL β-glucan for 24 h. At 24 h post-infection SVCV replication in ZF4 cells was evaluated by *q*PCR.

### RNA isolation and reverse transcription

Total RNA was isolated from spleen tissue of zebrafish and ZF4 cells with Trizol reagent (TaKaRa, Tokyo, Japan) following the manufacturer’s protocol. The extracted RNA was re-suspended in 30 μl RNase-free water then quantified with a BioTek Synergy™2 Multi-detection Microplate Reader (BioTek Instruments, Winooski, VT) and agarose gel electrophoresis. One microgram of total RNA was used for reverse transcription with Revert Aid™ Reverse Transcriptase (TaKaRa, Tokyo, Japan) according to the manufacturer’s instructions. The synthesized cDNA was stored at -20°C.

### Real-time quantitative PCR

Experimental methods about RT-*q*PCR reaction were conducted as previously described (34). The primers used in the experiment were listed in **Table 2**. Ribosomal protein s11 gene (*rps11*) was selected and used as the internal reference gene according to our previous work (35, 36), and the data were statistically analyzed by 2^-ΔΔCT^ method.

### 16S ribosomal RNA gene sequencing

At the end of the 3-weeks feeding period, the digesta of adult zebrafish were collected 4 h after the last feeding. The digesta were collected under aseptic conditions. The digesta samples from the 6 fish were pooled as a replicate. DNA was extracted from each pooled sample using a Fast DNA SPIN Kit for Soil (MP Biomedicals), according to the manufacturer’s instructions. The 16s V3–V4 region was amplified by using the primers U341F (5′-CGGCAACGAGCGCAACCC-3′) and U806 (5′-CCATTGTAGCACGTGTGTAGCC-3′). 16S *r*RNA gene sequencing was performed at the Realbio Genomics Institute using the Illumina Miseq platform. Microbiota sequencing data in this study are available from the National Center for Biotechnology Information (NCBI) under accession number PRJNA876409.

### Statistical analysis

All data were performed using GraphPad Prism 8 software (GraphPad Software Inc. CA, USA). All data were expressed as mean ± SEM. Differences between treatments were evaluated by Student’s *t*-test. The test of Log-rank (Mantel-Cox) was used to analyze survival rate after SVCV challenge. When *P* values were less than 0.05, the difference was considered significant.

## Results

### Dietary β-glucan enhances antiviral resistance of zebrafish

**Figure 1:**
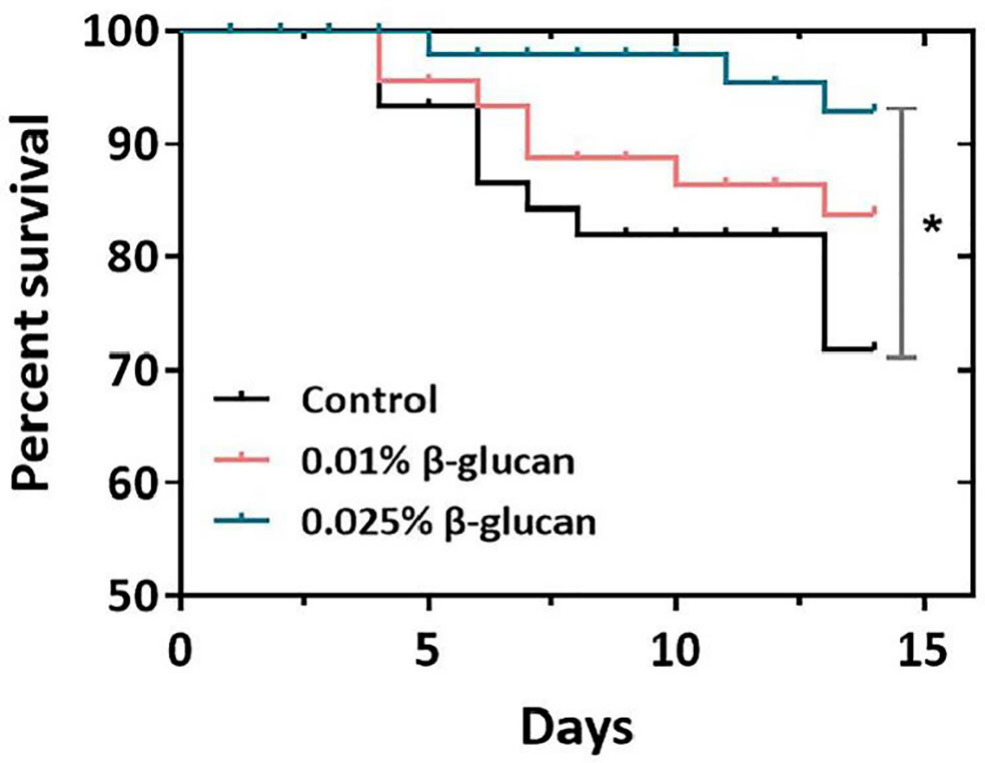
Effect of β-glucan on survival rates of zebrafish post SVCV infection (n = 3) (survival rates (%) over time, days 0-14). The asterisk denotes significant difference (*P* < 0.05) by Log-rank (Mantel-Cox) test.

As shown in **Figure 1**, the survival rate of zebrafish was calculated after challenge with 10^6^ TCID_50_/ml SVCV. Dietary supplementation of 0.025% β-glucan significantly increased survival rate of fish compared with control group (*P* < 0.05), while there was no significant difference between the 0.01% β-glucan group and control (*P* > 0.05). We also evaluated the effect of β-glucan on viral susceptibility of zebrafish larvae. Zebrafish larvae were treated with 0.025% and 0.05% (*m*/v) β-glucan by immersion and infected with SVCV. Results showed that β-glucan significantly inhibited viral replication in larvae (*P* < 0.01) (**Supplementary Figure 1**)

### Effect of β-glucan on antiviral immune response of zebrafish

**Figure 2:**
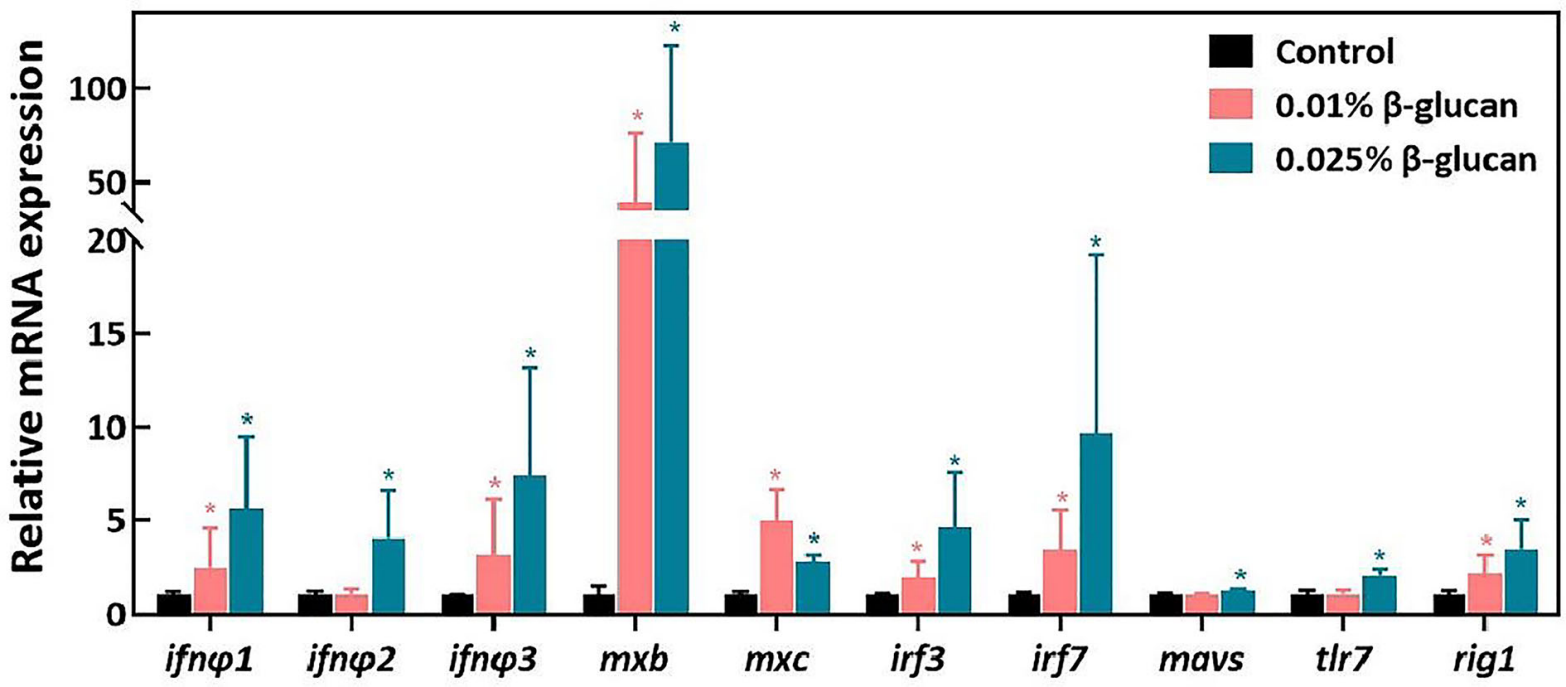
Relative mRNA expression of genes related to antiviral innate immune response in the spleen of zebrafish four days after SVCV challenge (n = 6). The expression of genes was expressed as fold of control group. Values represent the means ± SEM.*P < 0.05.

The expression of antiviral genes after SVCV challenge in zebrafish spleen are shown in **Figure 2**. Compared with the control group, the expression of type I IFN genes including *ifnφ1*, *ifnφ2*, *ifnφ3* and the expression of IFN-stimulated genes including *mxb* and *mxc* were significantly increased in the 0.025% β-glucan group (**Figure 2**, *P* < 0.05). In addition, 0.025% β-glucan group also significantly increased the expression of virus recognition receptors related genes including *tlr7*, *rig1* as well as the expression of downstream genes of IFN pathway such as *mavs*, *irf3* and *irf7* (**Figure 2**, *P* < 0.05). Similarly, 0.01% β-glucan supplementation also significantly increased the expression of *ifnφ1*, *ifnφ2*, *ifnφ3*, *mxb*, *mxc*, *irf3*, *irf7* and *rig1* (**Figure 2**, *P* < 0.05). Consistently, β-glucan treatment by immersion enhanced the expression of type I IFN pathway-related genes in zebrafish larvae after SVCV infection (**Supplementary Figure 2**).

### The antiviral effect of β-glucan is dependent on type I IFN response

**Figure 3:**
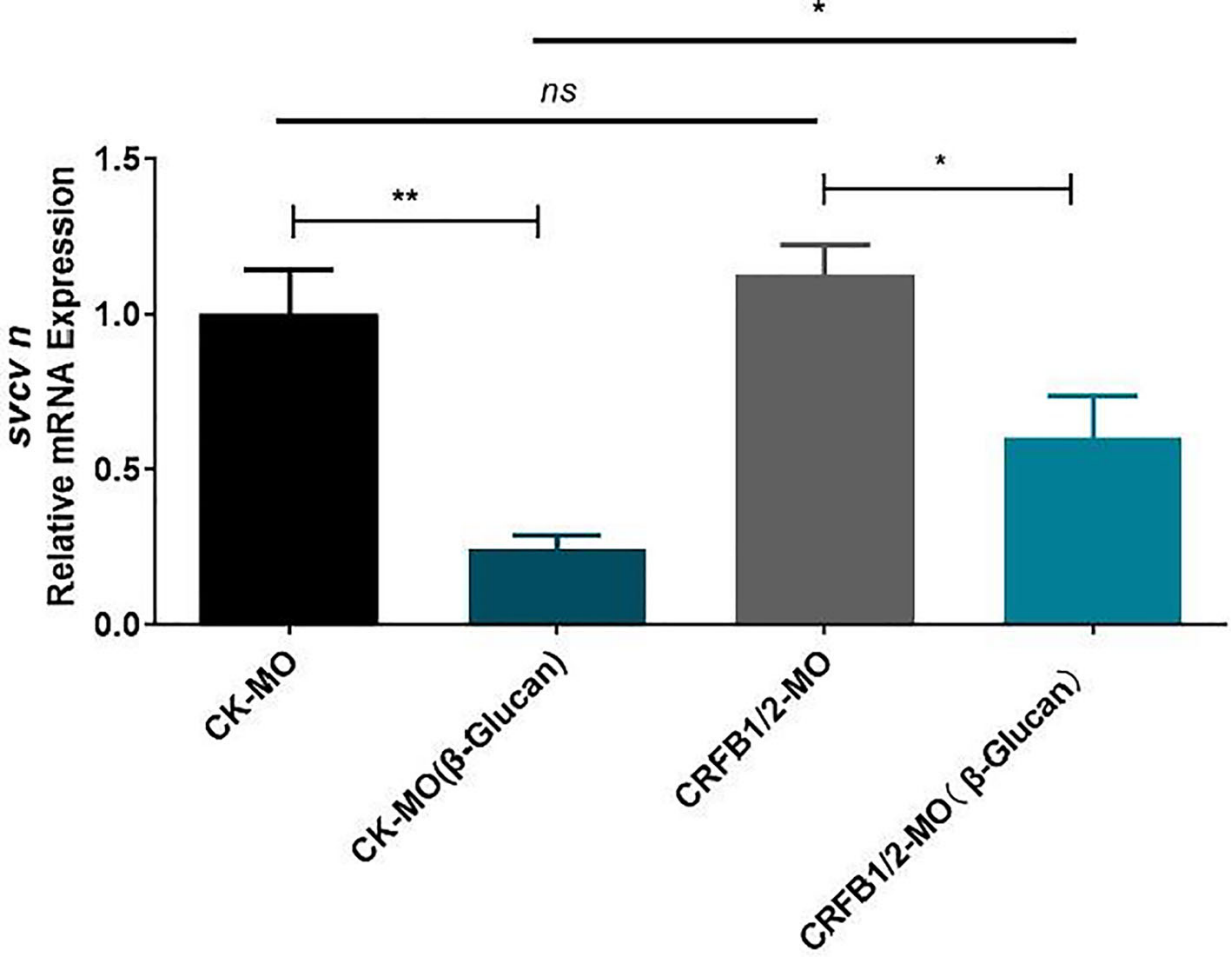
Effects of morpholino-mediated knockdown of IFN receptor subunits (CRFB1 and CRFB2) on the antiviral function of β-glucan in zebrafish larvae (n = 6). Values represent the means ± SEM. *P < 0.05, **P < 0.01, "ns" is a contraction of no significant.

The stimulation of genes in the IFN pathway by β-glucan in both adult and larval zebrafish suggest the involvement of IFN response in the antiviral function of β-glucan. To investigate the role of type I IFN response in the antiviral effect of β-glucan, the IFN receptor subunits CRFB1 and CRFB2 were knocked down in zebrafish larvae by vivo morpholino (**Supplementary Figure 3**). The results showed that while β-glucan still inhibited viral replication in zebrafish larvae with deficient IFN receptors, the inhibitory scale was significantly reduced (**Figure 3**), indicating that the mechanism of antiviral function of β-glucan is at least in part dependent on the stimulation of type I IFN antiviral immune response.

### β-glucan inhibits SVCV infection in ZF4 cells

**Figure 4:**
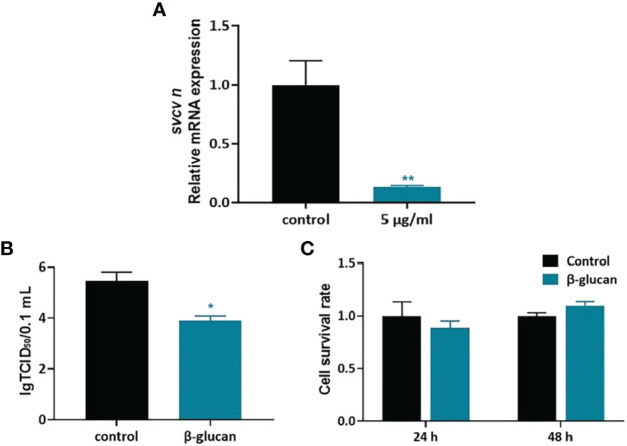
Antiviral effect of β-glucan in ZF4 cells. **(A)** Effects of β-glucan in the mRNA expression of SVCV N protein (n = 6); **(B)** Effects of β-glucan on the TCID_50_ of SVCV (n = 6); **(C)** Effects of β-glucan on cell viability (n = 6). Values represent the means ± SEM. *P < 0.05; **P < 0.01.

We further evaluated the antiviral effect of β-glucan in ZF4 cell model. ZF4 cells were treated with 1, 2.5, 5, 10, 20 μg/mL β-glucan for 24 h and then were infected by SVCV (**Supplementary Figure 4**). Viral replication in ZF4 cells was evaluated at 24 hpi. Results showed that 5 μg/mL β-glucan inhibited the replication of SVCV in ZF4 cells (**Figure 4A**, *P* < 0.05), which is consistent with the *in vivo* results. Moreover, compared with the control group, β-glucan significantly reduced the SVCV titer in the supernatant of ZF4 cells (**Figure 4B**, *P* < 0.05). We also evaluated the effect of β-glucan on cell viability. The results showed that 5 μg/mL β-glucan treatment for 24 and 48 h did not affect the survival of ZF4 cells (**Figure 4C**, *P* > 0.05).

### Effect of β-glucan on antiviral immune response of ZF4 cells

To eliminate the influence of viral replication on antiviral gene expression, viral RNA mimics polyriboinosinic polyribocytidylic acid (poly (I:C) was transfected into ZF4 cells after β-glucan treatment. The expression of genes related to the type I IFN signaling pathway was detected. Results showed that the expression of *ifnφ1*, *ifnφ2*, *ifnφ3*, *mxb*, and *mxc* after poly (I:C) stimulation was not significantly different in 5 μg/mL β-glucan compared with control (**Supplementary Figure 5**, *P* > 0.05), suggesting that β-glucan does not stimulate type I IFN antiviral response in ZF4 cells. The discrepancy of results in terms of IFN response in ZF4 cells and *in vivo* might be due to cell specific stimulation of IFN pathway by β-glucan in zebrafish.

### β-glucan enhances autophagy of ZF4 cells after viral infection

**Figure 5:**
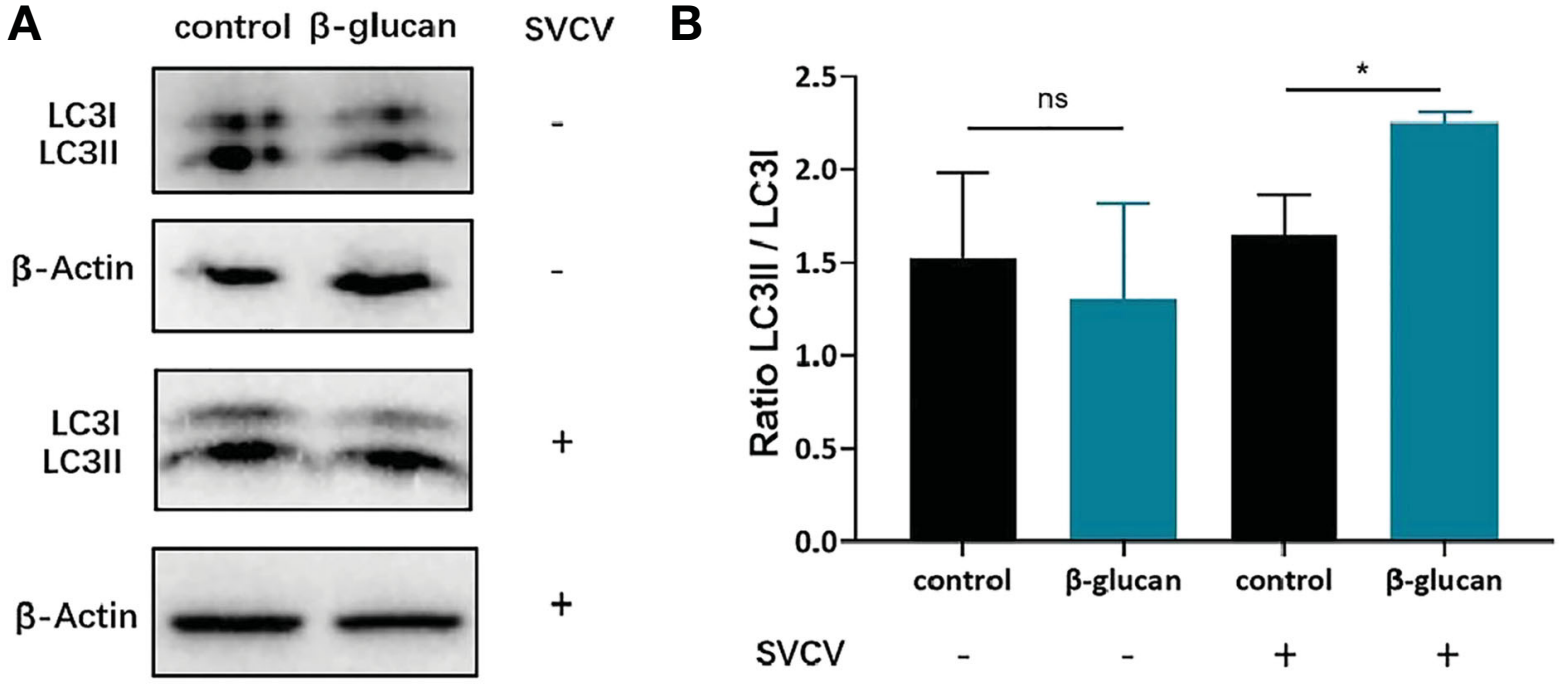
Effects of β-glucan on the LC3II/LC3I ratio post SVCV infection in ZF4 cells. **(A)** Western blot analysis of LC3 in ZF4 cells (n = 3); **(B)** LC3II/LC3I ratio in ZF4 cells were calculated by Image J software (n = 3). Values represent the means ± SEM. *P < 0.05, "ns" is a contraction of no significant.

LC3 is conjugated to phosphatidylethanolamine to form LC3-II after autophagy induction, which is conserved among vertebrates including fish. LC3-II/LC3-I ratio is commonly used to evaluate autophagy activation. In order to investigate the effect of β-glucan on autophagy formation in ZF4 cells, western blot for LC3 was performed. The results showed that β-glucan did not affect LC3-II/LC3-I ratio in non-infected cells, but significantly increased the ratio after SVCV infection (**Figures 5A, B**), indicating that β-glucan can enhance autophagy induction after viral infection.

**Figure 6:**
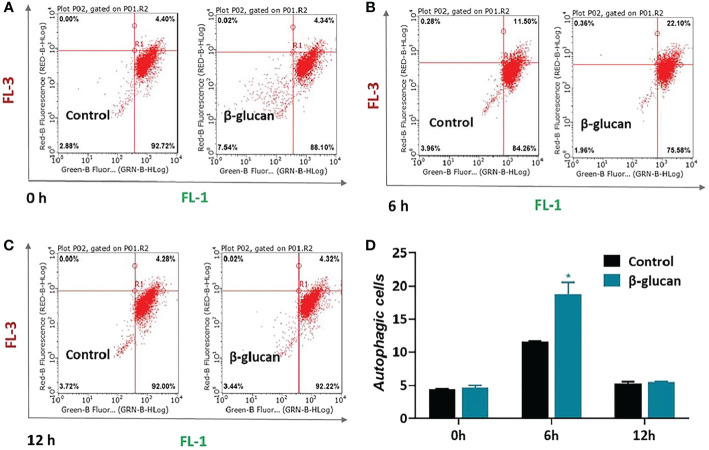
Effects of β-glucan on autophagy level in ZF4 cells. Autophagy was quantified by flow cytometry after AO staining. The flow cytometry results of control or β-glucan-treated ZF4 cells at 0 h **(A)**, 6 h **(B)**, and 12 h **(C)** post SVCV infection were exhibited; **(D)** Percentage of control or β-glucan-treated ZF4 cells positive for autophagosomes at different time points after viral infection. Values represent the means ± SEM. *P < 0.05 (n = 6).

Furthermore, autophagy was quantified by flow cytometry after AO staining. AO fluoresces green in whole cell but fluoresces red in autophagic vacuoles. The results showed that β-glucan significantly enhanced the autophagy levels of ZF4 cells 6 h after SVCV infection (**Figures 6B, D**, *P* < 0.05), while there was no significant difference in autophagy between β-glucan-treated and control cells at 0 h and 12 h post infection (**Figures 6A, C, D**, *P* > 0.05). This indicated that the β-glucan-mediated autophagy activation was transient and occurred in the early stage after viral infection.

**Figure 7:**
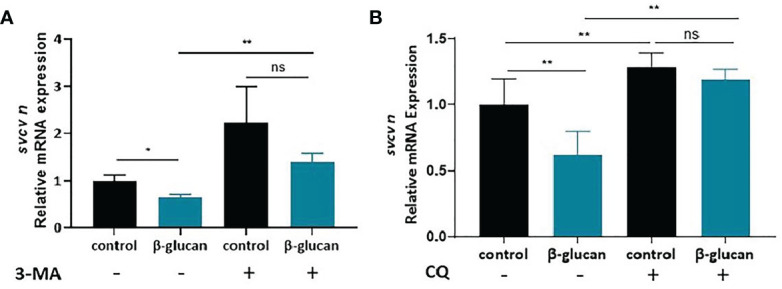
Effects of 3-MA **(A)** and CQ **(B)** on the antiviral effect of β-glucan in ZF4 cells (n = 6). Values represent the means ± SEM. *P < 0.05, **P < 0.01, "ns" is a contraction of no significant..

To investigate the role of autophagy in β-glucan-induced antiviral effect, we used 3-MA and CQ to inhibit autophagy. The results showed that pretreatment with both autophagy inhibitors blocked the antiviral effect of β-glucan in ZF4 cells (**Figures 7A, B**, **Supplementary Figures 6A, B**).

### The antiviral function of β-Glucan is independent of Myd88

**Figure 8:**
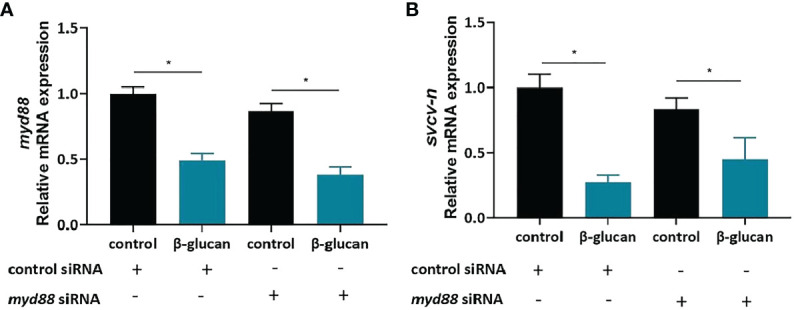
*Myd88* is not required for β-glucan to protect against SVCV infection. **(A)** The knockdown efficiency of *myd88* (n = 6); **(B)** Effects of β-glucan on the mRNA expression of SVCV N protein after siRNA knockdown of *myd88* (n = 6). Values represent the means ± SEM. *P < 0.05.

To gain insights into the potential receptors of β-glucan, we evaluated the role of Myd88 in the antiviral effect. siRNA of *myd88* was used to knock down *myd88* gene in ZF4 cells. The *myd88* gene was successfully knocked down by siRNA (**Figure 8A**). However, the viral replication was still significantly reduced in cells with deficient Myd88 (**Figure 8B**, *P* < 0.05), indicating that Myd88 was not required for β-glucan-mediated antiviral effect.

### Dietary β-glucan modulated the composition of gut microbiota

**Figure 9:**
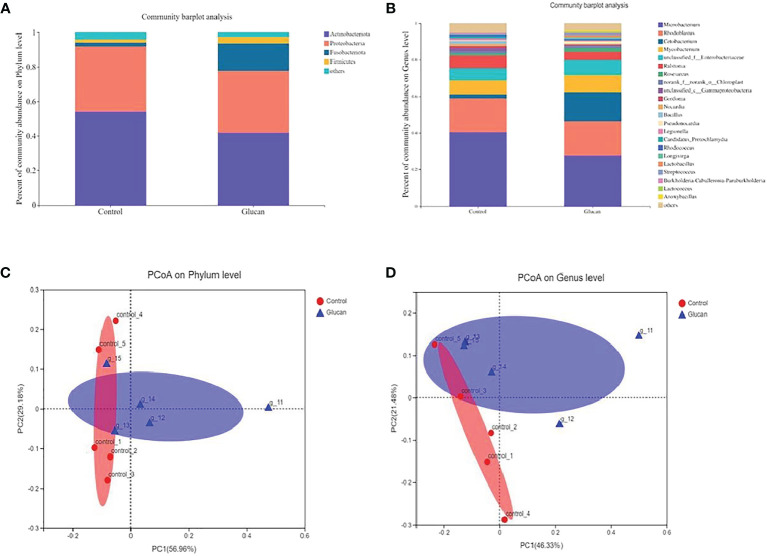
The composition of gut microbiota in zebrafish fed with or without 0.025% b-glucan diets. The composition of gut microbiota in zebrafish was analyzed using 16S rRNA sequencing analysis. **(A)** Relative abundance at the phylum level between the two groups; **(B)** Relative abundance at the genus level between the two groups; **(C, D)** Principal coordinate analysis (PCoA) of all samples by weighted UniFrac distance.

The result of α- diversity of gut microbiota was shown in **Supplementary Table 1**. The α-diversity of gut microbiota showed no significant difference between control group and β-glucan group. Compared to the control group, dietary β-glucan decreased the relative abundance of Actinobacteria and Proteobacteria while increased Fusobacteriota and Firmicutes abundance at the phylum level (**Figure 9A**). At the genus level, the addition of β-glucan increased the abundance of *Cetobacterium* and *Lactobacillus* (**Figure 9B**, n = 6). PCoA analysis showed significant difference in the composition of gut microbiota between the control group and the 0.025% β-glucan group at the phylum and genus level (**Figures 9C, D**, n = 6).

## Discussion

Glucans play a very important role in aquaculture as immunostimulant to improve the performance and health of farmed animals. β-Glucan has been found to induce immunity in various cultured fish species and resistance against different pathogens has been reported (8, 37, 38). Research have shown that β-glucan dosages, quality, route, and time of administration and duration of treatments may influence the its beneficial effects on growth, survival, and immunity of fish (39). Medina-Gali et al. reported that injection of yeast β-glucan enhanced zebrafish immune responses against SVCV and increased zebrafish survival after challenge with SVCV (22). However, the effect of dietary β-glucan on antiviral resistance of fish has not been reported yet, and the underlying mechanism was largely unknown. In the present study we investigated the antiviral potential of dietary β-glucan in zebrafish. Survival rate was significantly increased in zebrafish fed diet supplemented with β-glucan compared with control. Moreover, dietary β-glucan enhanced the expression of genes related to the type I IFN pathway, including IFNs, IFN-stimulated genes, virus recognition receptors, and downstream genes of IFN pathway. To confirm the involvement of type I IFN response in the antiviral effect of β-glucan, we knocked down the IFN receptors to block the IFN antiviral immunity. Zebrafish has two groups of type I IFNs: group I (IFNφ1 and IFNφ4) and group II (IFNφ2 and IFNφ3), which were recognized by different heterodimeric receptors, CRFB1/CRFB5 and CRFB2/CRFB5, respectively (33). Therefore, simultaneous knockdown of CRFB1 and CRFB2 can block the IFN antiviral immunity in zebrafish. We found that knockdown of the two IFN receptor subunits partially blocked the antiviral effect of β-glucan in zebrafish larvae, suggesting that the mechanism of antiviral function of β-glucan at least in part involves IFN response. The partial blockage of antiviral phenotype by the morpholinos might be due to incomplete deletion of the IFN receptors (**Supplementary Figure 3**). Alternatively, it suggests that in addition to type I IFN, other antiviral pathways are involved in β-glucan-mediated antiviral effect, which deserves further investigation.

We found that 5 μg/mL β-glucan can significantly reduce SVCV replication in ZF4 cells, which is consistent with the *in vivo* results. We further investigated the activation of type I interferon signaling pathway by β-glucan in ZF4 cells. We used poly (I:C) to avoid the confounding factors associated with viral replication that can affect the expression of antiviral genes. To our surprise, the results showed that β-glucan did not induce IFN signaling in ZF4 cells. In contrast with results in ZF4 cells, β-glucan enhanced type I interferon signaling pathway *in vivo*, both in the spleen of adult zebrafish (dietary supplementation) and in zebrafish larvae (immersion). This discrepancy might be due to that β-glucan stimulates IFN signaling in specific cell types of zebrafish, which does not include fibroblast cells such as ZF4 cell line.

In mammalian studies, several receptors have been reported to be involved in β-glucan recognition and binding, including TLR2, Dectin-1, and CR3 (complement receptor 3) (8, 40–42). Dectin-1 is a member of the C-type lectin family. It has been suggested that the pattern recognition receptors of β-glucan in fish may be other members of C-type lectin family (43). β-glucan enhances *tlr2* gene expression in both european eel (*Anguilla anguilla*) and zebrafish (44), and enhanced carp (*Cyprinus carpio*) *tlr3* gene expression (45). This suggests that the zebrafish TLR2 receptor might be a receptor for β-glucan. CR3 receptor is a promiscuous pattern recognition receptor recognizing many ligands including β-glucan. The involvement of CR3 as β-glucan receptor in fish has been less studied. Increased expression of CR3 gene was observed in the intestine of Atlantic salmon after oral intake of purified β-glucan, suggesting its involvement in the recognition of glucan (46). We used siRNA to knock down Myd88, which is a key adaptor protein for TLR receptors. Knockdown of Myd88 did not affect the antiviral effect of β-glucan in ZF4 cells, suggesting that β-glucan receptor is not a TLR in zebrafish. The recognition receptor(s) of β-glucan in fish, which mediates the downstream antiviral effects, deserves further investigation.

Autophagy can play either antiviral or proviral roles, depending on the virus (47). Previous studies showed that SVCV infection induces autophagy in both ZF4 cells and zebrafish larvae, and autophagy inhibits SVCV infection (29, 30). Consistently, our results showed that SVCV infection induced autophagy in ZF4 cells. On top of it, we found that β-glucan enhanced autophagy after SVCV infection, and the antiviral effect of β-glucan was blocked *in vitro* by autophagy inhibitors, implicating the involvement of autophagy induction in the antiviral effect of β-glucan in ZF4 cells. The influence of β-glucan on autophagy has been reported previously. Yeast β-glucan inhibited autophagy of liver cancer cells (48), while β-glucan derived from *Agaricus bisporus* showed autophagy induction activity in zebrafish (49). The effect of β-glucan on autophagy might depend on the source, doses, and the cells. Notably, β-glucan treatment only enhanced autophagy of ZF4 cells after SVCV infection, but did not induce autophagy in non-infected cells, suggesting a synergistic effect of SVCV infection and β-glucan on autophagy induction. This was different from previous studies (48, 49), in which the effect on autophagy was observed at basal condition. The relationship between autophagy and type I IFN antiviral immunity has been studied recently in mammalian virus infection model (50), but related knowledge is largely unknown about fish viruses. The link between autophagy and IFN response in SVCV-zebrafish infection model deserves further investigation.

Reports reveal that the gut microbiota plays an important role in regulating the human immune system and improving gut health (51). Recently, studies have shown that the gut microbiota also regulates viral infection (52, 53). The intestinal microbiota may exert antiviral function by regulating the antiviral immunity of host. Ichinohe et al. (2011) (54) found that the microbiota is crucial for the immune response against influenza virus in mice. To evaluate the response of intestinal microbiota to the intake of β-glucan, the composition of intestinal microbiota in zebrafish was analyzed by sequencing the 16S *r*RNA gene. Dietary 0.025% β-glucan decreased the relative abundance of Actinobacteria and Proteobacteria while increased Fusobacteriota and Firmicutes abundance at the phylum level. At the genus level, the addition of β-glucan increased the abundance of *Cetobacterium* and *Lactobacillus*. The bacterial taxa in the microbiota responsible for the antiviral function deserve further investigation. Notably, we observed that β-glucan can improve the expression of intestinal HIF-1α (hypoxia-inducible factor-1α) (**Supplementary Figure 7**). HIF has been recognized as an important regulator of intestinal homeostasis (55). In our previous study, stimulation of intestinal HIF-1α was proved to have beneficial effect on the microbiota composition (34). Therefore, the influence of β-glucan on intestinal microbiota of zebrafish might be attributable to its effect on HIF-1α.

In summary, the present study revealed that the dietary supplementation of β-glucan can enhance the antiviral ability of zebrafish. β-glucan stimulated the type I IFN signaling in both adult and larval zebrafish after SVCV infection. Mechanistic study by morpholino-mediated knockdown of IFN receptors confirmed the involvement of type I IFN pathway in the antiviral effect of β-glucan. Furthermore, we found that β-glucan inhibited SVCV infection in ZF4 cells. While β-glucan did not affect antiviral immune response in ZF4 cells, it induced autophagy of the cells after viral infection. Lastly, we found that dietary β-glucan altered the intestinal microbiota of zebrafish, and the β-glucan-associated microbiota might also contribute to the antiviral function.

## Data availability statement

The original contributions presented in the study are included in the article/**Supplementary Material**. Further inquiries can be directed to the corresponding authors.

## Ethics statement

All experimental procedures were performed in accordance with the “Guidelines for Experimental Animals” of the Ministry of Science and Technology (Beijing, China). The study was reviewed and approved by the Feed Research Institute of the Chinese Academy of Agricultural Sciences Animal Care Committee under the auspices of the China Council for Animal Care. All efforts were made to minimize suffering.

## Author contributions

HL and YL: formal analysis, data curation, writing original draft preparation and revising the draft. ML, WZ and JC: investigation, interpretation of data. ZZ: analysis and resources. YLY: analysis and resources. CR: research design, data curation, funding acquisition, revising the draft, revised and edited the manuscript. ZGZ: research design, data curation, funding acquisition and revising the draft. All authors contributed to the article and approved the submitted version.

## Funding

This work was funded by grants from the National Key R&D Program of China (2018YFD0900400) and National Natural Science Foundation of China (31925038, 32122088).

## Conflict of interest

The authors declare that the research was conducted in the absence of any commercial or financial relationships that could be construed as a potential conflict of interest.

## Publisher’s note

All claims expressed in this article are solely those of the authors and do not necessarily represent those of their affiliated organizations, or those of the publisher, the editors and the reviewers. Any product that may be evaluated in this article, or claim that may be made by its manufacturer, is not guaranteed or endorsed by the publisher.
