# Corrigendum: The effect and underlying mechanism of yeast β-glucan on antiviral resistance of zebrafish against spring viremia of carp virus infection

**DOI:** 10.3389/fimmu.2024.1357392

**Published:** 2024-02-19

**Authors:** Hui Liang, Yu Li, Ming Li, Wei Zhou, Jie Chen, Zhen Zhang, Yalin Yang, Chao Ran, Zhigang Zhou

**Affiliations:** ^1^ Key Laboratory for Feed Biotechnology of the Ministry of Agriculture and Rural Affairs, Institute of Feed Research, Chinese Academy of Agricultural Sciences, Beijing, China; ^2^ Laboratory of Gene Therapy, Department of Biochemistry, College of Life Sciences, Shaanxi Normal University, Xi’an, China; ^3^ Sino-Norway Joint Lab on Fish Gut Microbiota, Institute of Feed Research, Chinese Academy of Agricultural Sciences, Beijing, China

**Keywords:** β-glucan, zebrafish, SVCV, antiviral immunity, gut microbiota

In the published article, there was an error in the legend for **Figure 3**, Effects of morpholino-mediated knockdown of IFN receptor subunits (CRFB1 and CRFB2) on the antiviral function of β-glucan in ZF4 cells (n =6).] as published.

The corrected legend appears below.

**Figure 3:**
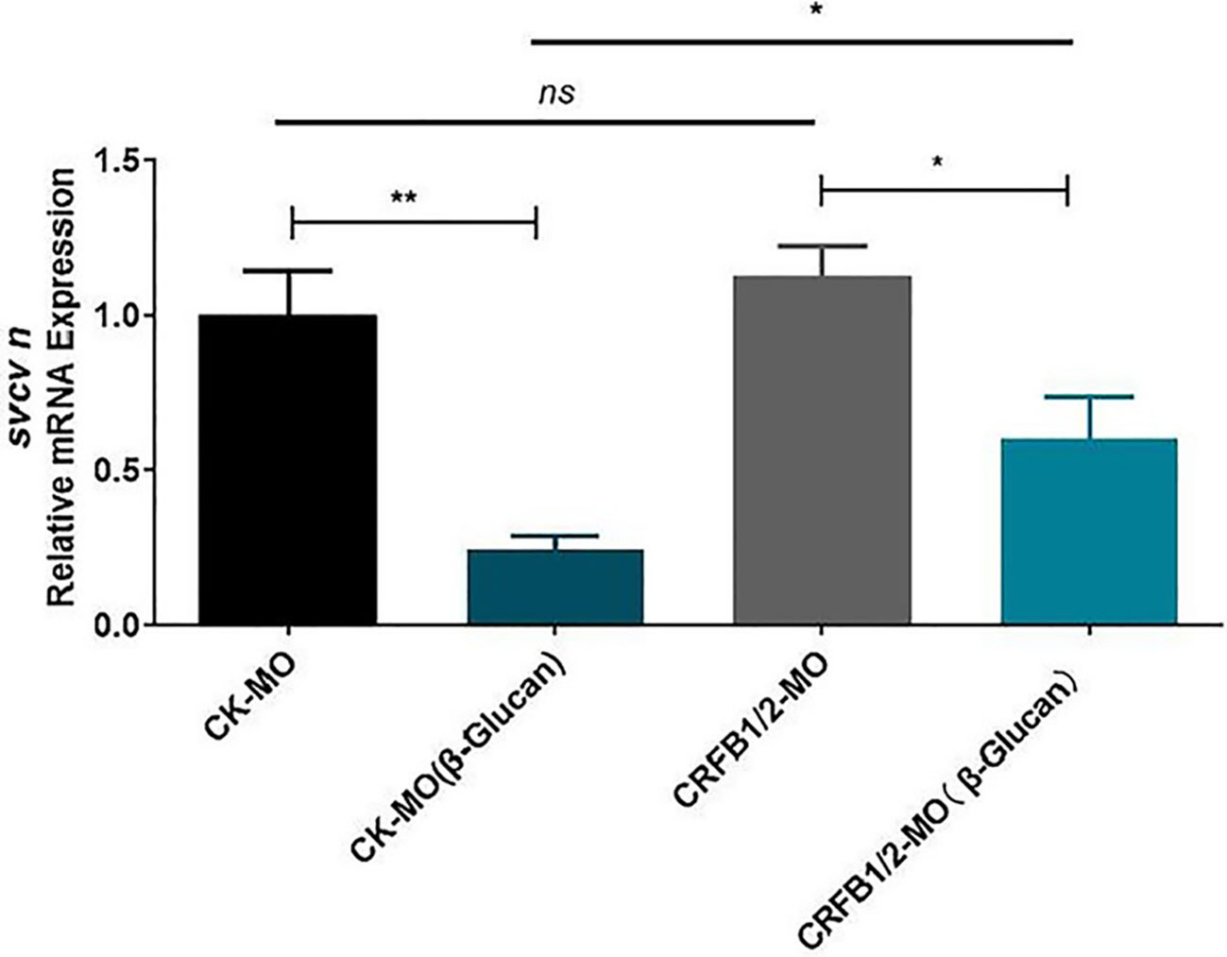
Effects of morpholino-mediated knockdown of IFN receptor subunits (CRFB1 and CRFB2) on the antiviral function of β-glucan in zebrafish larvae (n =6).

In the published article, there was a spelling error. A correction has been made to the **Results** section, paragraph 9. This sentence previously stated: “The antiviral function of β-Glucan is ndependent of Myd88.”

The corrected sentence appears below.

“The antiviral function of β-Glucan is independent of Myd88.”

The authors apologize for these errors and state that this does not change the scientific conclusions of the article in any way. The original article has been updated.

