# Supplementary material for: The effect and underlying mechanism of yeast β-glucan on antiviral resistance of zebrafish against spring viremia of carp virus infection
Source: Front Immunol. 2022 Nov 3;13:1031962. doi: 10.3389/fimmu.2022.1031962 (PMC9669391; doi:10.3389/fimmu.2022.1031962)
Supplement: Supplementary file 1 [file DataSheet_1.docx]

**Supplemental Methods**

**Tissue sampling and western blot analysis**

Zebrafish gut tissues were homogenized in ice-cold HBSS buffer mixed with 1 mM PMSF and phosphatase inhibitors. Protein was quantified by the BCA protein assay kit (Beyotime). Immunoblot analysis was performed as described previously (1). The primary antibodies include antibodies against GAPDH (SAB2708126, 1:2000; Sigma) and HIF-1α (Bioworld, BS3514, 1:1000) were used in the experiment.

**Supplemental Figures**


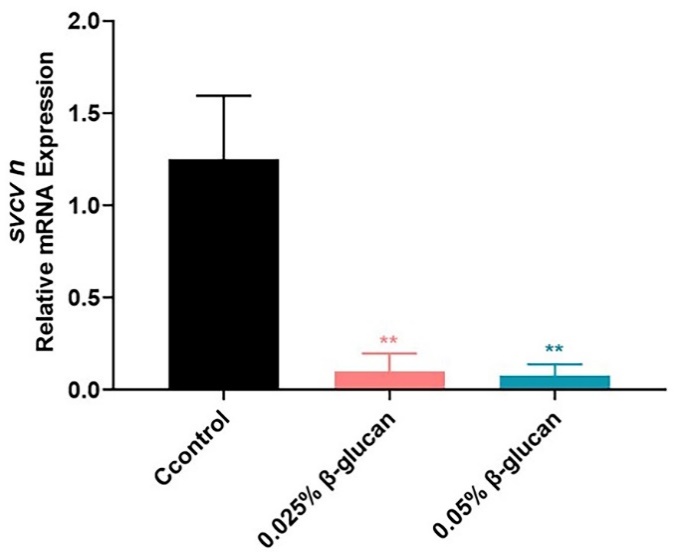


**Supplementary Figure 1** Effects of β-glucan treatment on the mRNA expression of SVCV N protein in zebrafish larvae at 48 h post SVCV infection. All values are expressed as mean ± SEM (n = 4), ***P* < 0.01*.*


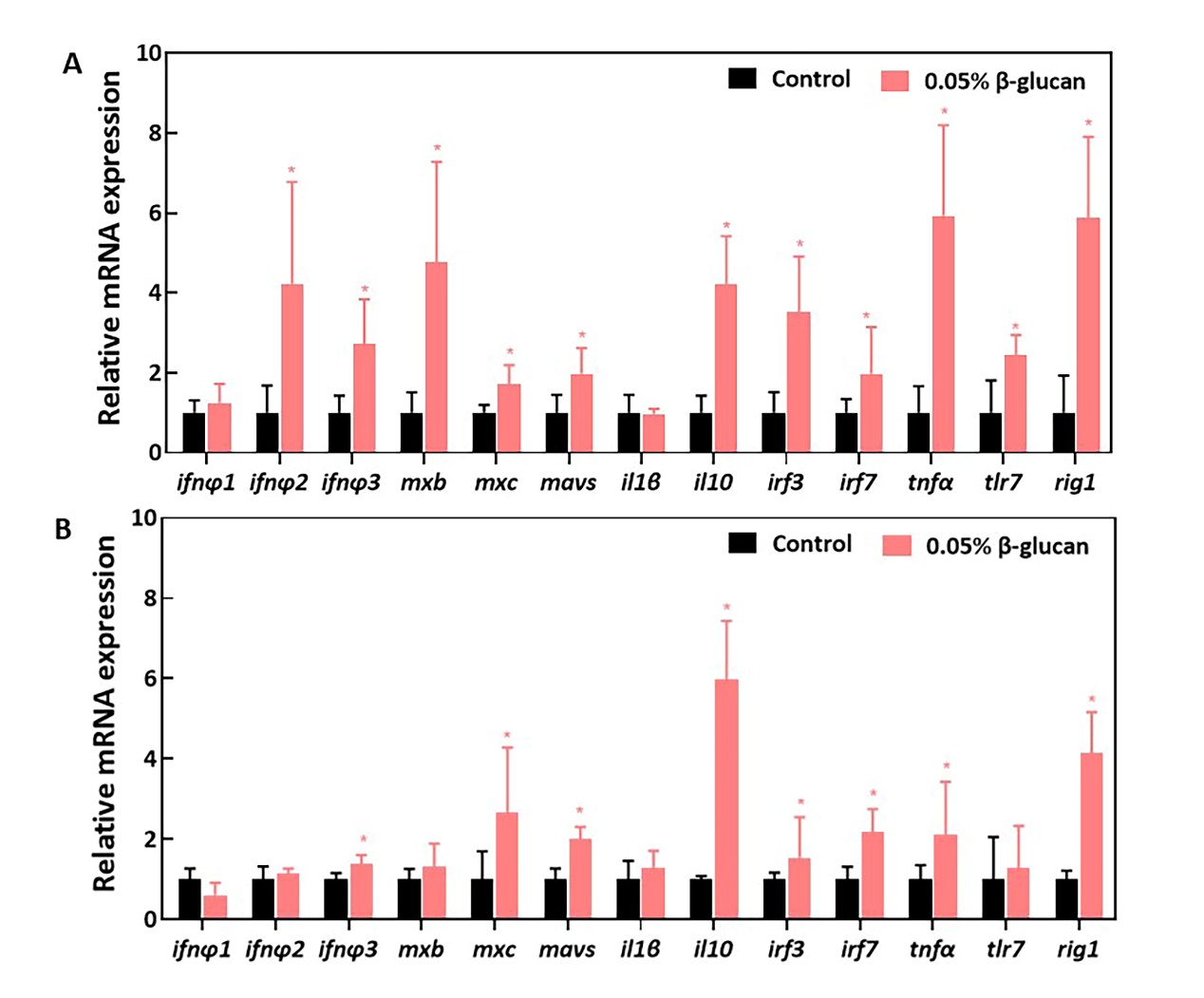


**Supplementary Figure 2** Relative mRNA expression of antiviral genes in zebrafish larvae 24 h **(A)** and 48 h **(B)** after SVCV challenge (n = 4). Values represent the means ± SEM.* *P*< 0.05.
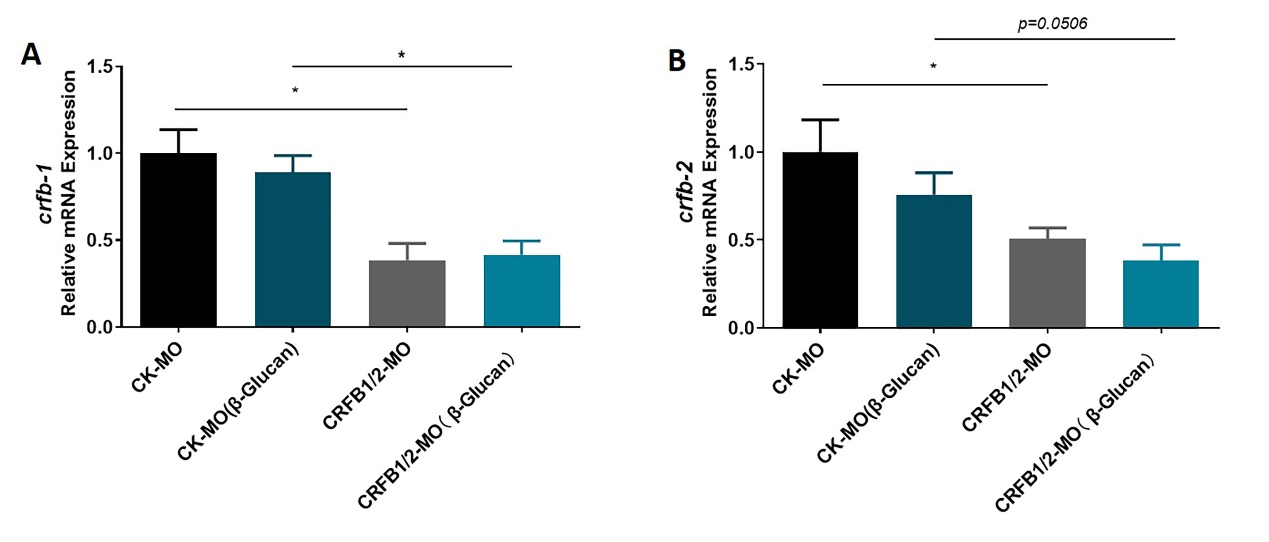


**Supplementary Figure 3** Detection of knockdown efficiency of CRFB1 **(A)** and CRFB2 **(B)** (n = 6). The relative expression of properly spliced transcripts was evaluated by *q*PCR. Values represent the means ± SEM. * *P*< 0.05.


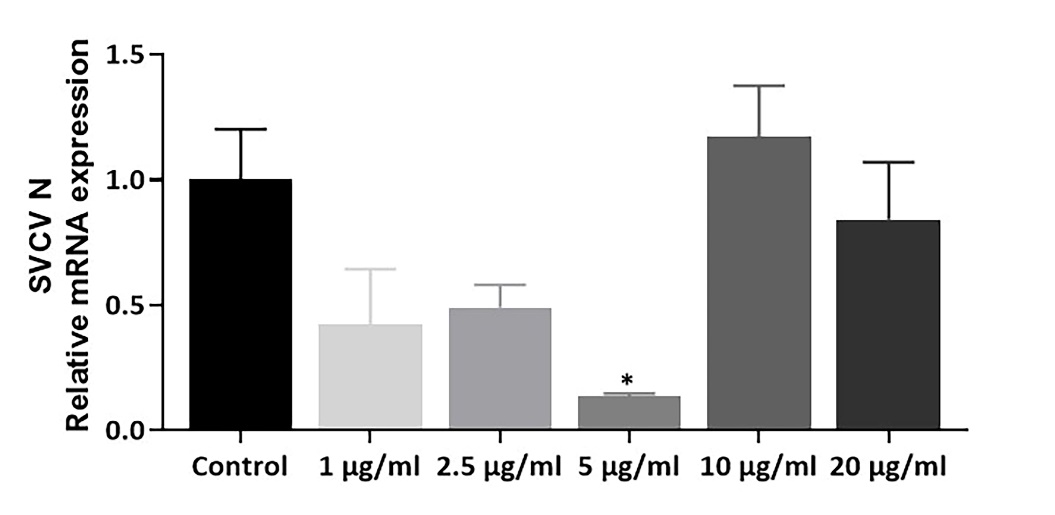


**Supplementary Figure 4** Antiviral effect of β-glucan in ZF4 cells. (n = 6).


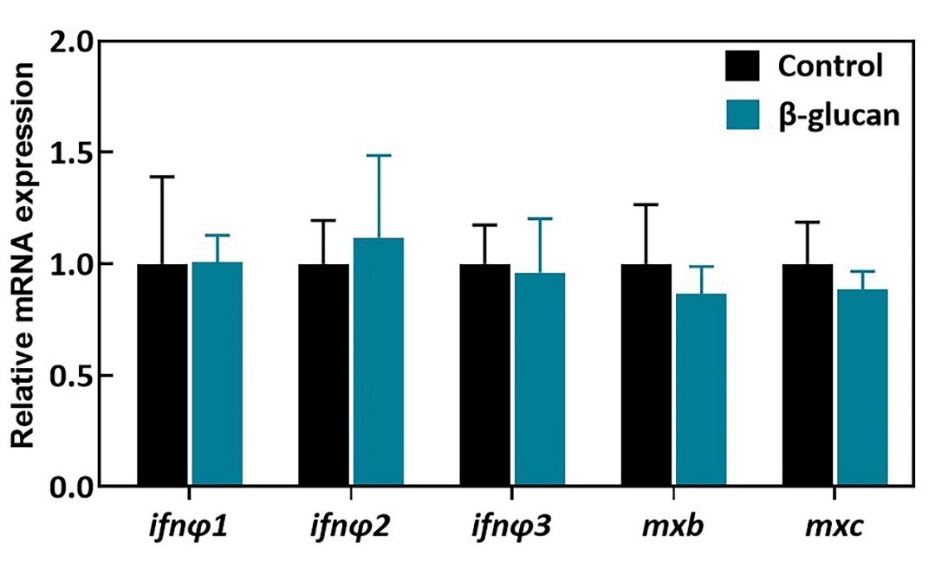


**Supplementary Figure 5** Effects of β-glucan on the mRNA expression of type Ⅰ IFN related genes in ZF4 cells after poly (I:C) stimulation (n = 6). Values represent the means ± SEM. * *P* < 0.05.


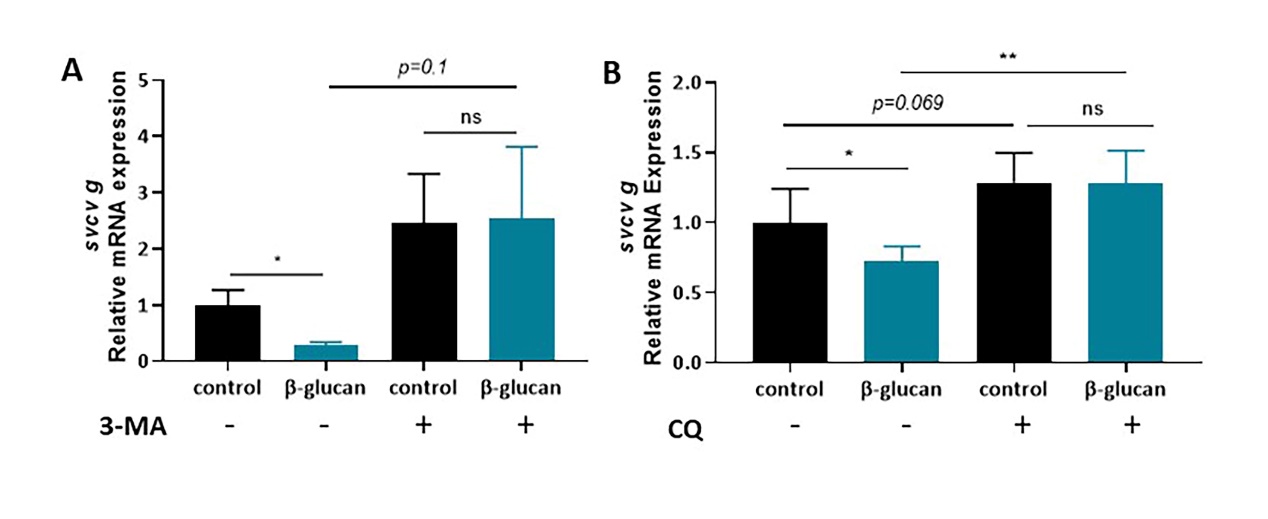


**Supplementary Figure 6** Effects of 3-MA (A) and CQ (B) on the antiviral effect of β-glucan in ZF4 cells. Values represent the means ± SEM. * *P* < 0.05.


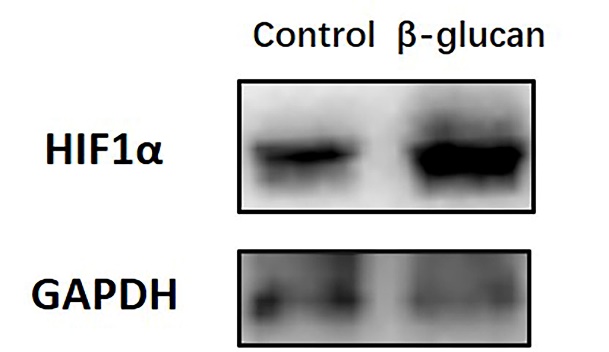


**Supplementary Figure 7** A representative western blotting showing the expression of HIF-1α in the intestine of zebrafish fed control or β-glucan diet.

**Supplementary TABLE 1 Effects of β-glucan on the α-diversity of gut microbiota of zebrafish.**

|  | **Shannon** | **Simpson** | **ACE** | **Chao** |
| --- | --- | --- | --- | --- |
| **Control** | 2.2114 ± 0.39266 | 0.25117 ± 0.10146 | 250.7 ± 22.839 | 248.86 ± 24.273 |
| **β-Glucan** | 2.1863 ± 0.31305 | 0.23411 ± 0.085001 | 272.85 ± 28.321 | 269.84 ± 26.235 |

Values are expressed as the mean t SEM, n = 5. OTU, operational taxonomic unit; Shannon, Shannon diversity index; Simpson, Simpson's diversity index; ACE, ACE index; Chao, Chao index.

**References**

1.Zhang Z, Ran C, Ding QW, Liu HL, Xie MX, Yang YL, Xie YD, Gao CC, Zhang HL, Zhou ZG. Ability of prebiotic polysaccharides to activate a HIF1α-antimicrobial peptide axis determines liver injury risk in zebrafish. Commun Biol (2019) 2: 274.
